# Supplementary material for: Computational and Preclinical Analysis of 2-(4-Methyl)benzylidene-4,7-dimethyl Indan-1-one (IPX-18): A Novel Arylidene Indanone Small Molecule with Anti-Inflammatory Activity via NF-κB and Nrf2 Signaling
Source: Biomedicines. 2023 Feb 27;11(3):716. doi: 10.3390/biomedicines11030716 (PMC10045539; doi:10.3390/biomedicines11030716)
Supplement: Supplementary file 1 [file biomedicines-11-00716-s001.zip › biomedicines-2124689-supplementary.pdf]

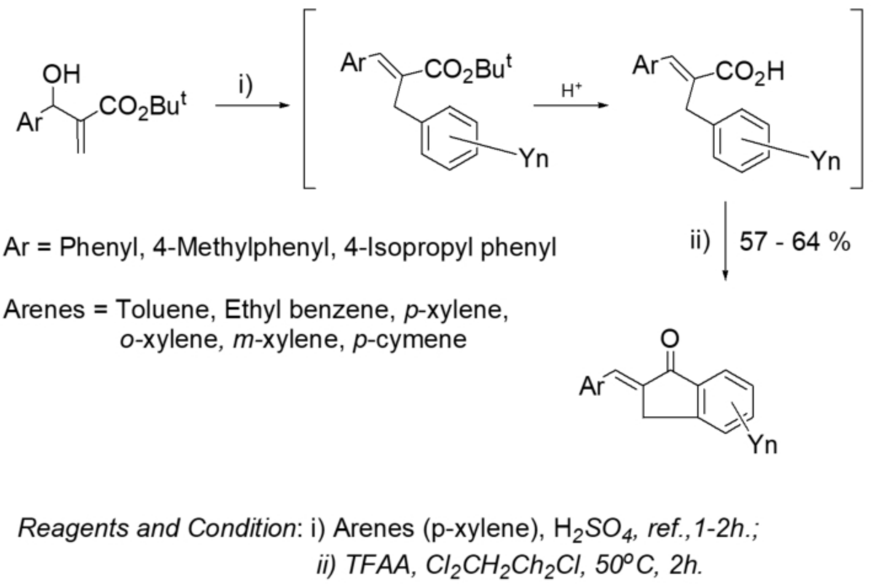

Figure S1. Schematic synthesis of IPX-18.

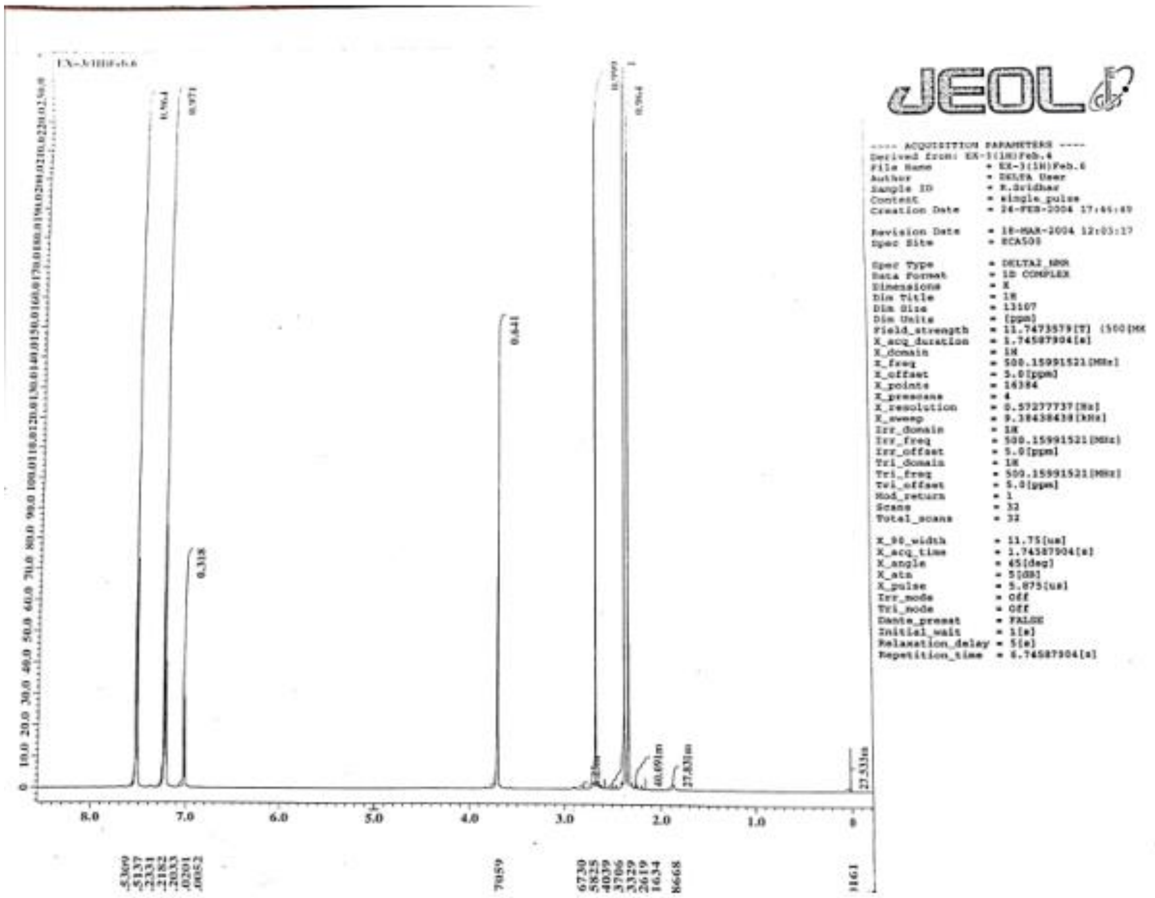

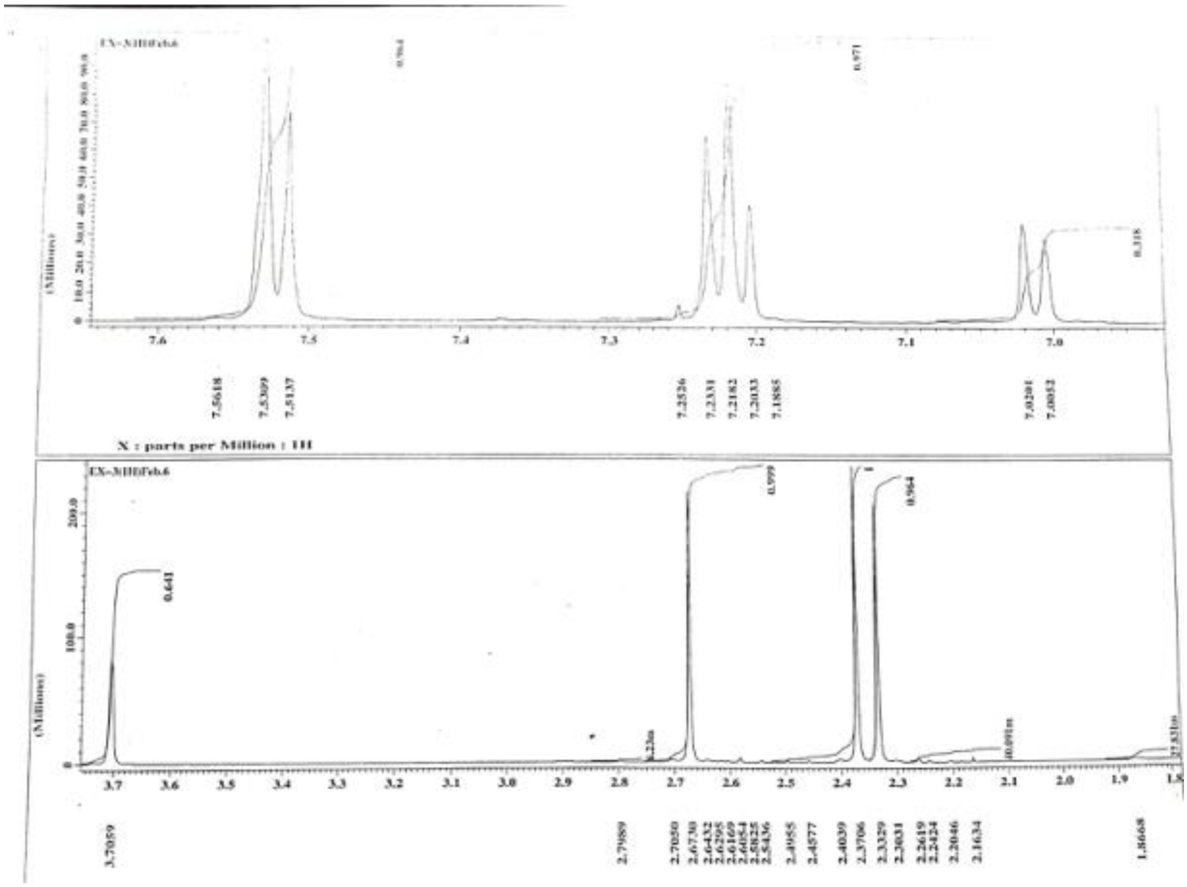
(Millions)
7.6
7.5
7.4
7.3
7.2
7.1
7.0
7.5618
7.5309
7.5137
7.2526
7.2331
7.2182
7.2033
7.1885
7.0201
7.0052
X : parts per Million : 1H
(Millions)
200.0
100.0
0.641
0.999
0.964
3.7
3.6
3.5
3.4
3.3
3.2
3.1
3.0
2.9
2.8
2.7
2.6
2.5
2.4
2.3
2.2
2.1
2.0
1.9
3.7059
2.7989
2.7050
2.6730
2.6432
2.6295
2.6169
2.6054
2.5825
2.5436
2.4955
2.4577
2.4039
2.3706
2.3329
2.3031
2.2619
2.2424
2.2046
2.1634
1.8668

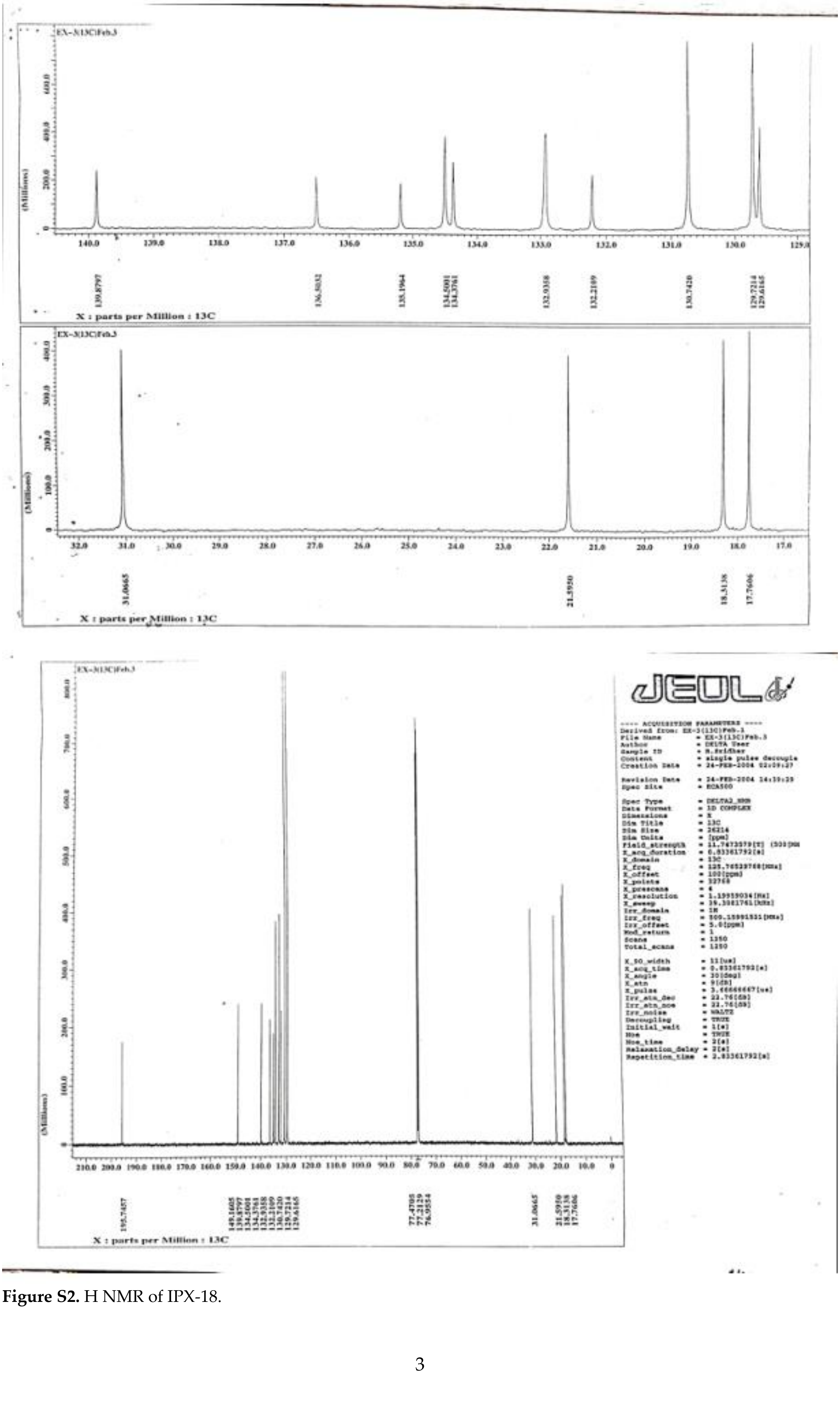

Figure S2. H NMR of IPX-18.

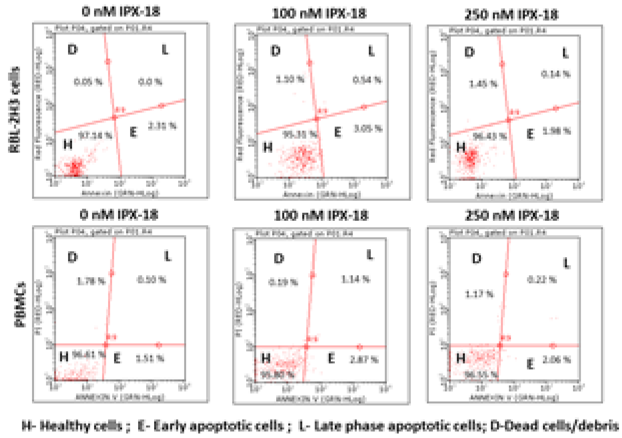

Figure S3. Representative graphs of the Annexin V assay in RBL-2H3 cells and PBMCs when treated with IPX-18 for 24 h. Results indicate no evidence of increment in the early or late apoptotic population in both cell types when treated with 100 nM or 250 nM IPX-18.

Supplementary video 1.mp4

Video S1. Simulation trajectory video shows the binding stability of IPX frame-by-frame.
